# Improving consenting practise in ENT surgery – measures that lead to effective change

**DOI:** 10.1186/1753-6561-6-S4-O34

**Published:** 2012-07-09

**Authors:** E Ridyard, V Varadarajan

**Affiliations:** 1 University of Manchester, UK

## Introduction

To measure consenting standards of common ENT procedures in a foundation trust department and implement effective measures to improve and standardise consenting practice.

## Methods

Consenting standards were compiled from ENT UK / BAOHNS patient leaflets for tonsillectomy, grommet insertion, septoplasty, rigid oesophagoscopy, functional endoscopic sinus surgery (FESS) and mastoidectomy. Prospective analysis of complications documented on consent forms was performed (n = 56). Deficient areas were raised amongst the department and pre-prepared stickers were produced for use on consent forms. A second prospective data cycle (n=59) was collected and analysed using chi-squared testing.

## Results

Improvements were seen in the vast majority of complications consented for. Statistically significant improvements were measured for septoplasty (“cosmetic change” (p = 0.05), “teeth numbness” (p = 0.0010)) and mastoidectomy (“dizziness” (p = 0.01), “tinnitus” (p = 0.05), “ear dressing reaction” (p = 0.001)). Some percentage decreases were seen for grommet insertion (“infection” (-10%)) and rigid oesophagoscopy (“perforation” (-25%)).

## Conclusions

A wide variety of consenting practice was measured. Improvement was seen in the vast majority of complications consented for. New doctors starting midway during the second data cycle may be responsible for the reduction in some standards. Sticker use was non-mandatory, and may be made mandatory to further improve standards.

